# Evaluation of biomechanical properties on partial and complete epitendinous suture in human cadaver flexor tendon repair

**DOI:** 10.1186/s13018-021-02645-6

**Published:** 2021-08-12

**Authors:** Thepparat Kanchanathepsak, Wilarat Wairojanakul, Sorasak Suppaphol, Ittirat Watcharananan, Panithan Tuntiyatorn, Tulyapruek Tawonsawatruk

**Affiliations:** 1 Hand and Microsurgery Unit, Department of Orthopaedics, Faculty of Medicine Ramathibodi Hospital, Mahidol University, 270 Rama VI Road, Ratchathewi, Bangkok, 10400 Thailand; 2 Chakri Naruebodindra Medical Institute, Faculty of Medicine Ramathibodi Hospital, Mahidol University, Samutprakan, Thailand

**Keywords:** Partial epitendinous suture, Complete epitendinous suture, Flexor tendon repair

## Abstract

**Objective:**

This study was designed to compare the ultimate tensile strength and force to 2 mm gap formation among 50% partial, 75% partial, and complete circumferential epitendinous suture with a combination of 4-strand core suture in human cadaver flexor tendon.

**Materials and methods:**

Forty-five flexor tendons from four soft human cadavers were used to evaluate the biomechanical property among 50% partial, 75% partial, and complete circumferential epitendinous suture with a combination of 4-strand core suture.

**Results:**

The force to 2 mm gap of complete epitendinous was significantly greater than partial epitendinous suture (*P* < 0.05); however, there was no difference between 50% partial and 75% partial epitendinous suture (*P* > 0.05). For the ultimate strength, there was no significant difference between partial and complete epitendinous suture (*P* > 0.05). The partial epitendinous was approximately 60% of the complete epitendinous suture in force to 2 mm gap and also 70% of complete epitendinous suture in ultimate tensile strength with a combination of core sutures.

**Conclusions:**

The complete epitendinous suture showed better ultimate tensile strength and force to 2 mm gap compared with a partial 50% and 75% epitendinous suture. However, in some clinical scenario which the complete epitendinous suture is not possible to perform, the authors suggested only partial epitendinous suture with 50% circumference is recommended as the additional epitendinous repair up 75% circumference cannot provide any mechanical benefit to the repaired site.

## Introduction

Flexor tendon injury is a common problem in hand surgery, and its treatment with a good result is a continuing challenge. The standard treatment of flexor tendon repair is composed of core suture and epitendinous suture, using many different techniques. The ideal repaired tendon should have adequate strength for early mobilization. This can promote intrinsic healing and prevent peritendinous adhesion.

It has been reported in several studies that at least 4 to 6 strands of core suture were preferred for sufficient tensile strength to allow early motion [1–4]. Biomechanics studies demonstrated that adding epitendinous suture improved strength, prevented gap formation, and provided a smooth repaired surface [3, 5–9].

Normally, the epitendinous suture method is circumferential suturing around the tendon. In some situations, the repair sites are located near the tendon insertion such as flexor tendon injury zone 1 or beneath the unresectable pulley, particularly the A2 and A4 pulleys. These injury sites are difficult for rotating or flipping the backside of the tendon and performing the complete circumferential epitendinous suture. Therefore, our hypothesis is using the standard core suture with the partially epitendinous suture would be sufficient to provide the tensile strength for early rehabilitation.

The goal of this study is to compare the ultimate tensile strength, force to 2 mm gap formation, and stiffness among partially circumferential of 50%, 75%, and complete 100% epitendinous suture.

## Materials and methods

Forty-five flexor tendons from four soft human cadavers, three right hands and three left hands, were harvested including two males and two females with an average age of 78 years (72–83). All of the flexor digitorum profundus tendon (FDP), flexor digitorum superficialis tendon (FDS) (except little finger due to smaller size compared with the others), and flexor pollicis longus tendon (FPL) were collected in this study. The flexor tendons were harvested at the level of 5 cm proximal to wrist crease and distal to A4 pulley for FDP and FDS tendons while FPL tendon was harvested distal to A2 pulley. The dissected tendons were wrapped in the saline-soaked gauze and stored at − 20 °C. The study was approved by the ethics committee of our institute.

**Fig. 1:**
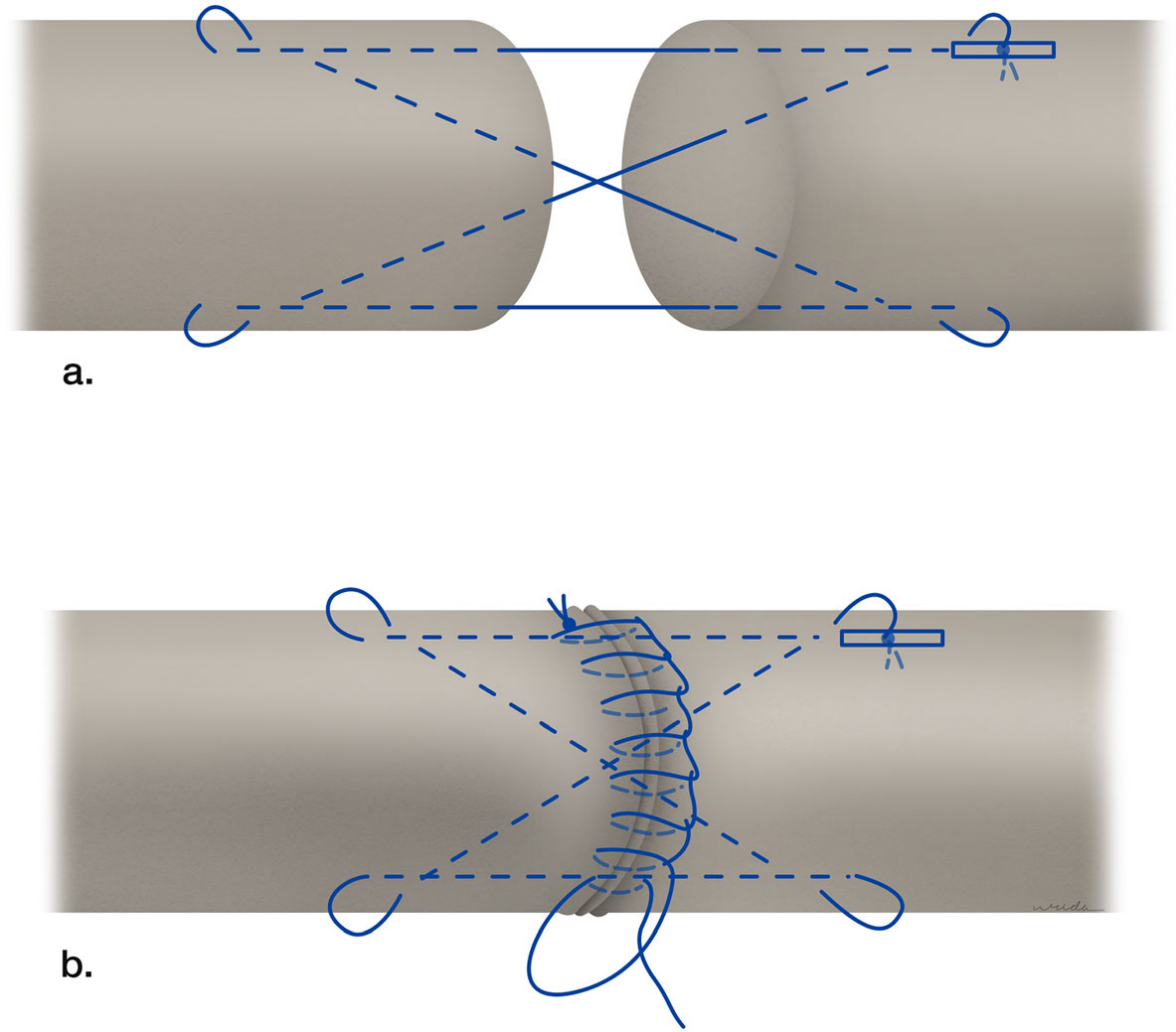
Illustration of suture techniques. **a** The 4-strand cruciate core suture. **b** The running locked epitendinous suture

On the day of experiment, all of the flexor tendons were thawed to room temperature and then simple randomly assigned into 3 groups, 15 tendons per group. The tendon was transected at the mid-length using a no. 15 surgical blade. There was no significant difference in area of tendon cut among 3 groups (group 1, 11.09 ± 0.87 mm^2^; group 2, 11.46 ± 0.97 mm^2^; and group 3, 12.03 ± 1.10 mm^2^, *p* > 0.05). Both sides of the transected surface of the tendon were marked in four quadrants as 25% each by circumference. The transected tendon was repaired by 4-strand cruciate technique which was originally described by McLarney et al. [10]. The 3-0 Nylon (Dafilon®, B.Braun Surgical, S.A., Rubi. Spain) was used as suture material (Fig. 1).

**Fig. 2:**
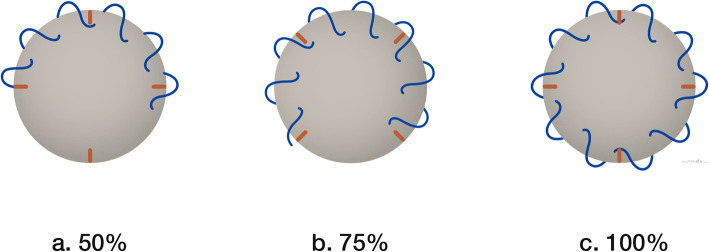
Description of the running locked epitendinous suture in cross-sectional view: **a** 50% circumferential, **b** 75% circumferential, and **c** 100% circumferential suture

In group 1, the epitendinous suture was repaired circumferentially for 50% using the running locked technique by 5-0 nylon (50C). In group 2, the epitendinous suture was repaired circumferentially for 75% using the running locked technique by 5-0 nylon (75C), and in group 3, the epitendinous suture was repaired completely for 100% circumferential using the same technique as former groups (100C) (Fig. 2).

The 4-strand core suture was performed with a 10-mm purchase on each side of the tendon edge, whereas the epitendinous suture was performed using a 2-mm purchase in length, depth, and interval. All sutures were performed by the same surgeon. All the suture knots were tied outside using one surgical throw and three simple throws.

The tendon biomechanics were tested using a Universal testing machine (SHIMADZU, EZ-S, Kyoto, Japan) with a 500-Newton (N) load cell. The proximal and distal ends of the repaired tendons were held with the grippers, 5 cm of length on each side. A 2 N preload was applied to all tendons then starting with initial load of 5 N. The force was increasing by 5 N with a distraction rate of 5 mm/min. The force was stopped at the point of 2 mm gap formation and then continued until failure, either suture breakage or pullout from the repaired site. The force at the failure of the tendon was measured as ultimate tensile strength (N). The force which produced 2 mm gap formation (N), the ultimate tensile strength, and stiffness (N/mm) were recorded.

### Statistical analysis

Statistical testing was analyzed using GraphPad Prism 8.4.2 software (GraphPad Software Inc., La Jolla, California). The normality of continuous data was tested using the Kolmogorov–Smirnov test. Continuous variables including the ultimate tensile strength, the force to produce a 2-mm gap, and the stiffness were normally distributed and presented as the mean ± SEM. The differences among groups for each parameter were analyzed using the ANOVA test. The post-test analysis was determined using Bonferroni’s multiple comparison test. The *P* value < 0.05 was considered to be statistically significant for all statistical tests.

## Results

**Fig. 3:**
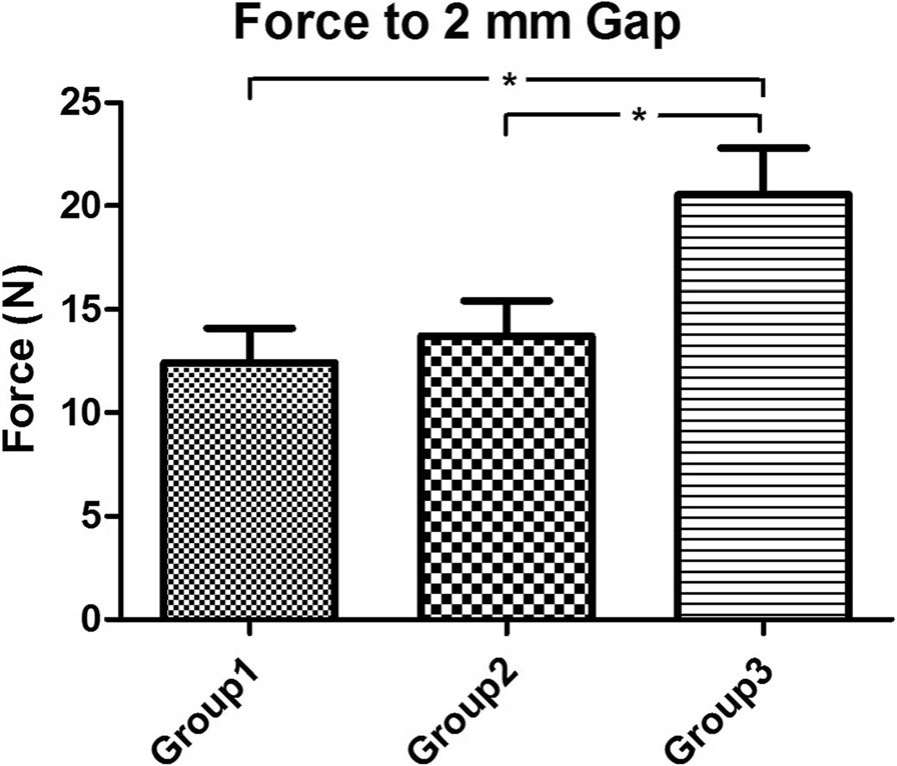
The mean force to 2 mm gap formation (N) among three groups. The partial epitendinous suture group (groups 1 and 2) was significantly lower than the complete epitendinous group (group 3) by post hoc analysis, *P < 0.05

**Fig. 4:**
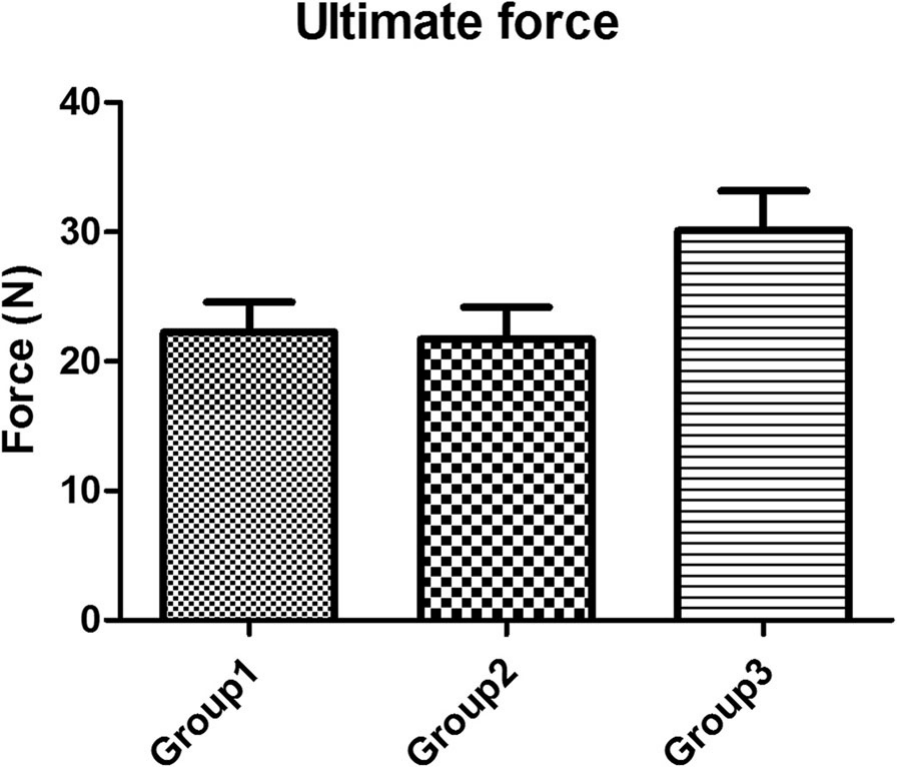
The ultimate tensile strength (N) showed highest in the complete epitendinous suture (group 3); however, there were no significant differences among the three groups (*P* > 0.05)

**Fig. 5:**
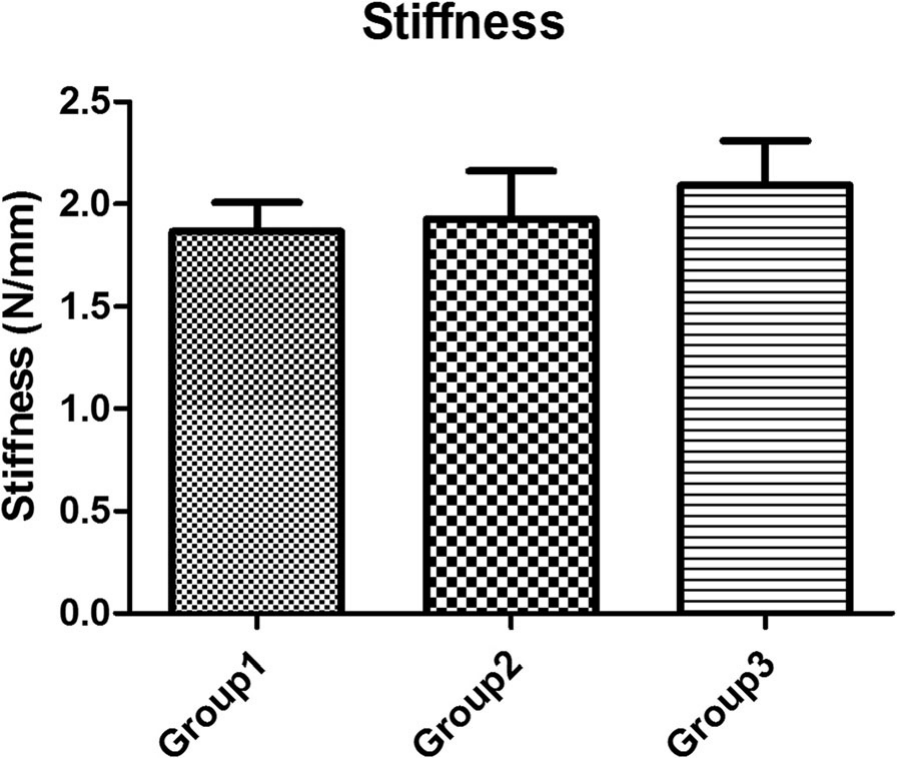
The mean stiffness (N/mm) showed no significant differences among the three groups (*P* > 0.05)

The mean forces to 2 mm gap formation between the 50C and the 75C group (12.4 ± 1.7 N and 13.7 ± 1.7 N, respectively) were not significantly different (*P* > 0.05). Whereas the force to 2 mm gap of the 50C and 75C were significantly lower than the 100C group (20.6 ± 2.3 N) (*P* < 0.05), for which partially epitendinous suture was approximately 60% of the complete epitendinous suture (Fig. 3). The highest ultimate tensile strength was in the 100C group (30.1 ± 3.1 N) followed by the 50C (22.2 ± 2.3 N) and the 75C (21.7 ± 2.5 N); thus, the partially epitendinous suture is approximately 70% of the ultimate tensile strength compared to complete epitendinous suture. However, there were no significant differences among the three groups (*P* > 0.05) (Fig. 4). The mean stiffness of the 50C (1.9 ± 0.1 N/mm), 75C (1.9 ± 0.2 N/mm), and 100C (2.1 ± 0.2 N/mm) groups did not have significant differences (*P* > 0.05) (Fig. 5). All tendons failed as a result of suture pullout from the repaired site.

## Discussion

It is accepted that the standard tendon repair is composed of core and epitendinous sutures. Peripheral epitendinous suture had been developed for several decades, and many studies have shown improving repaired tendon strength, reduced gap formation between tendon ends, and provided smooth surface of the repaired site [3, 5–9]. However, in some situations, the circumferential epitendinous suture is technically limited for example on the dorsal side of tendon near the insertion or beneath unresectable pulleys.

Our study showed the force to 2 mm gap formation from three different percentages of the epitendinous sutures in human cadaveric flexor tendons. It was found that the 100C is significantly stronger than the partial 50C and 75C epitendinous sutures. However, no significant difference in ultimate tensile strength and stiffness between partial and complete epitendinous suture was noted.

Ansari et al. reported the ultimate force of the partial epitendinous suture compared to complete epitendinous sutures. The results showed that a palmar half of an epitendinous repair improved 20% ultimate strength of a 4-strand core suture and prevented early gap formation. However, the ultimate force of failure of partial epitendinous suture was lower than that of a complete epitendinous with core suture [11]. The authors compared the suture technique only the palmar half and complete epitendinous suture in a porcine tendon, whereas our study compared the suture technique in the human cadaveric tendon in different percentages of epitendinous suture.

Similarly, Takeuchi et al. used artificial tendon roll to evaluate partial repair technique, and the results showed that complete circumferential had better strength and gap formation force than the partial half or three fourths of circumferential peripheral suture [5].

However, our study showed the partial epitendinous suture was approximately 60% of complete epitendinous suture in force to 2 mm gap and also 70% of complete epitendinous suture in ultimate tensile strength with combination of core suture that these outcomes resemble as the previous studied [11]. The results of ultimate tensile strength and force to 2 mm gap formation in this study are slightly lower than in the previous study probably because of different numbers of strands, suture material, suture techniques, types of testing tendons, and testing protocol [5, 6, 9, 11, 12]. Moreover, it is possible that the core suture with tension across the repaired site may be different from the previous studies [13].

From our study, the ultimate force and stiffness were not significant among the three different percentages of epitendinous sutures, which were different to the previous reports. We believe that the core suture contributed more in ultimate force and the stiffness of the repair. Meanwhile, epitendinous repair affected more in force to 2 mm gap as in the different percentage of epitendinous repair could lead to unbalance force across the repair site which in resulted in lower force to 2 mm gap in partial repair.

All of our repaired tendon failed via suture pullout mode, which may be explained by performing the cruciate core suture without locking loop according to what was originally described by McLarney et al. [10] where mechanical properties of locking loop showed greater tensile strength and reduced gap formation than grasping loop [14, 15].

The strength of this study is that it was performed on human cadaver and randomized by various flexor tendons in the hand including the FDP, FDS, and FPL tendons, which is representative of the entire tendon possibly injured in clinical practice. The previous studied had been conducted using porcine tendon or artificial tendon roll which may not be relevant to clinical situations. The limitations are that this was an in vitro study without gliding resistance from a pulley system and soft tissue surrounding the tendon that might inhibit early motion and result in gap formation [16, 17]. Furthermore, our study used a linear loading test model and no cyclic loading test, which is not compatible with the physiologic loading of the flexor tendon, particularly in early active rehabilitation protocol [9, 18–20].

## Conclusion

A combination of 4 strand core suture with complete epitendinous suture had better ultimate tensile strength and force to 2 mm gap than partial epitendinous suture. There was no significance difference in ultimate tensile strength and force to 2 mm gap between 50% partial and 75% partial of epitendinous suture. According to our results, in some clinical scenario in which the complete epitendinous suture is not possible to perform with complete circumference, partial epitendinous suture with 50% circumference is recommended, as the additional epitendinous repair up 75% circumference cannot provide any mechanical benefit to the repaired site. The operative time should be faster, and the technique is simpler with partial 50% circumferential repair.

## Data Availability

All data generated or analyzed during this study are included in this published article.

## Abbreviations

N: Newton
FDP: Flexor digitorum profundus
FDS: Flexor digitorum superficialis
FPL: Flexor pollicis longus
